# TRPM7/RPSA Complex Regulates Pancreatic Cancer Cell Migration

**DOI:** 10.3389/fcell.2020.00549

**Published:** 2020-07-08

**Authors:** Thibaut Lefebvre, Pierre Rybarczyk, Clara Bretaudeau, Alison Vanlaeys, Rémi Cousin, Sylvie Brassart-Pasco, Denis Chatelain, Isabelle Dhennin-Duthille, Halima Ouadid-Ahidouch, Bertrand Brassart, Mathieu Gautier

**Affiliations:** ^1^Laboratoire de Physiologie Cellulaire et Moléculaire – UR-UPJV 4667, UFR Sciences, Université de Picardie Jules Verne (UPJV), Amiens, France; ^2^Service d’Anatomie et Cytologie Pathologiques, CHU Amiens-Picardie, Amiens, France; ^3^UMR CNRS 7369 Matrice Extracellulaire et Dynamique Cellulaire (MEDyC), Université de Reims Champagne Ardenne (URCA), Reims, France

**Keywords:** pancreatic ductal adenocarcinoma, elastin-derived peptides, transient receptor potential melastatin-related 7, ribosomal protein SA, cell migration

## Abstract

Pancreatic ductal adenocarcinoma (PDAC) is a malignancy with a very poor prognosis due to highly metastatic profile. Cell migration is an essential step of the metastatic cascade allowing cancer cells to spread toward target tissues. Recent studies strongly suggest that bioactive elastin peptides, also named elastokines or elastin-derived peptides (EDPs), are released in the extracellular microenvironment during tumoral remodeling of the stroma. EDPs stimulate cancer cell migration by interacting with their membrane receptor, ribosomal protein SA (RPSA). Others membrane proteins like ion channels are also involved in cancer cell migration. It has been recently shown that the transient receptor potential melastatin-related 7 (TRPM7) channel regulates PDAC cell migration and invasion. The objective of this work was to study the effect of EDPs on TRPM7 channel in human pancreatic cancer cells. We showed that EDPs promote MIA PaCa-2 cell migration using Boyden chamber assay. Cells transfected with a siRNA targeting TRPM7 were not able to migrate in response to EDPs indicating that TRPM7 regulated cell migration induced by these peptides. Moreover, EDPs were able to stimulate TRPM7 currents recorded by Patch-Clamp. Finally, we showed that TRPM7 channels and RPSA receptors are colocalized at the plasma membrane of human pancreatic cancer cells. Taken together, our data suggest that TRPM7/RPSA complex regulated human pancreatic cancer cell migration. This complex may be a promising therapeutic target in PDAC.

## Introduction

Pancreatic ductal adenocarcinoma (PDAC) represents 85–90% of all pancreatic cancer types. The incidence of PDAC is continuously increasing in such a way that PDAC is expected to be the second cancer in term of mortality in 2030 (Rahib et al., 2014). PDAC is characterized by an abundant desmoplastic stroma that participates to the formation of metastasis and chemoresistance. This remodeled stroma is a complex structure composed of extracellular matrix (ECM) proteins and various cell types. Cancer development is influenced by ECM components. ECM/cell interactions involve cell adhesion to extracellular macromolecules through cell surface receptors and lead to ECM degradation and bioactive ECM macromolecule fragments release, called matrikines. Elastin is the major component of elastic fibers, particularly abundant in elastic tissues such as arteries and lung. Its proteolysis by elastase-type proteinases (metalloproteinases, pancreatic elastase, leucocyte elastase) is linked to the genesis of several diseases affecting elastin-rich organs (Lohmann et al., 1994; Houghton et al., 2011). This degradation is known to unmask cryptic sites within the ECM and to release matrikines, termed elastin-derived peptides (EDPs) or elastokines. These EDPs display a wide range of biological activities, influencing cell migration (Senior et al., 1980; Da Silva et al., 2018), differentiation (Betre et al., 2006), proliferation, chemotaxis (Long et al., 1988; Da Silva et al., 2018), survival, tumor progression (Huet et al., 2002; Toupance et al., 2012; Donet et al., 2014; Brassart et al., 2019), angiogenesis (Robinet et al., 2005), aneurysm formation, and atherogenesis. Among all the EDPs described in the literature, two categories of EDPs were listed: VGVAPG, VAPG, VGVPG, VGAPG, (VGVAPG)n, and PGAIPG with the xGxxPG consensus sequence, and, AGVPGLGVG, AGVPGFGVG, GLGVGVAPG, and GFGVGAGVP with the xGxPGxGxG consensus sequence. *In vivo* study showed that xGxPGxGxG peptides like AG-9 promote tumor progression to a greater extent than do xGxxPG peptides like VG-6. These results were confirmed by *in vitro* studies in proliferation assays, migration assays, adhesion assays, proteinase secretion studies, and pseudotube formation assays to investigate angiogenesis (Da Silva et al., 2018). The set of these biological properties regulated by AG-9 and VG-6 peptides involves a lactose-insensitive receptor, the ribosomal protein SA (RPSA) (Brassart et al., 2019). Mecham et al. (1989) were the first to report the 37/67-kDa laminin receptor to bind elastin. The 37/67-kDa laminin receptor, RPSA, also known as 37LRP, 67LR, ICAS, LAMBR, LAMR1, LBP, LBP/p40, LRP, LRP/LR, NEM/1CHD4, SA, lamR, and p40, is ubiquitously expressed. It provides cellular adhesion to the basement membrane. The major forms described for RPSA were 37-, 53-, and 67-kDa forms but several groups have reported the presence of additional high-molecular-weight (HMW) forms of 32, 37, 45, 53, 55, 67, 80, and >110-kDa. The nature of conversion of the 37-kDa form to higher molecular weight species remains poorly understood (DiGiacomo and Meruelo, 2016). The RPSA receptor is located in the nucleus [association with nucleolar pre-40S ribosomes, small nucleolar ribonucleoproteins (snoRNPs), chromatin, histones], in the cytosol (ribosomal component; co-localize with actin and cytoskeletal stress fibers) and at the cell surface. It mediates cell proliferation, adhesion, and differentiation. It was reported to enhance tumor cell invasion and adhesion as well as angiogenesis, key steps in tumor progression. Recent findings have shown that RPSA is involved in the maintenance of cell viability through apoptotic evasion, allowing tumor progression (Vania et al., 2019). The green-tea-derived polyphenol, (−)-epigallocatechin-3-gallate (EGCG), is a small molecule that was reported to affect cell behavior through RPSA binding and cytoskeletal alterations. EGCG inhibitory effect appears to be blocked by RPSA antibodies, which do not trigger the same effects, indicating that the polyphenol may act agonistically or allosterically (DiGiacomo and Meruelo, 2016). The functional domain responsible for the anti-cancer activity of EGCG may be located in the 10 amino acid sequence of RPSA, IPCNNKGAHS (Fujimura et al., 2012).

The RPSA has been very recently shown to be overexpressed in PDAC tissues in relation-enhanced cell invasion, metastasis, and poor prognosis (Wu et al., 2019). We recently showed that PDAC cell migration and invasion are regulated by the transient receptor potential melastatin-related 7 (TRPM7) channel expression (Rybarczyk et al., 2012, 2017). TRPM7 expression is also increased in PDAC tissues in relation with poor prognosis (Rybarczyk et al., 2012; Yee et al., 2015). TRPM7 is a non-selective cation channel fused with a kinase domain at its C-terminus (Nadler et al., 2001; Runnels et al., 2001). As both RPSA and TRPM7 are overexpressed and regulate cancer cell migration, it is tempting to speculate that these two biomarkers could interact in PDAC. The aim of this study is to determine how TRPM7 and RPSA regulate enhanced PDAC cell migration induced by EDPs.

## Materials and Methods

### Cell Culture

Human pancreatic cancer cell line MIA PaCa-2 (ATCC CRL-1420) was used for this study. This cell line was derived from a poorly differentiated tumor which corresponds to a grade 3 PDAC (Deer et al., 2010). MIA PaCa-2 cells were cultured in Dulbecco’s modified Eagle’s medium (Gibco) supplemented with 10% FCS (Lonza). Cells were trypsinized once a week using trypsin-EDTA (Sigma-Aldrich) and incubated at +37°C in a humidified atmosphere with 5% CO_2_.

### Elastin Peptides

VG-6 and AG-9 peptides were purchased from Proteogenix (Schiltigheim, France). EGCG was purchased from Enzo Life Sciences. Rabbit anti-TRPM7 and anti-RPSA antibodies were purchased from Abcam.

### Cell Migration

Migration tests were performed in 8-μm pore size polyethylene terephthalate membrane cell culture inserts (BD FALCONTM Cell Culture Inserts, BD Biosciences). The upper compartment was seeded with 4.10^4^ cells in FCS-free growth medium with or without different synthetic elastin peptide concentration (10^–9^ to 10^–7^ M) for 24 h at +37°C. The lower compartment was also filled with FCS-free growth medium. Thus, migration assays were performed without addition of chemoattractant. After incubation, cells were washed by phosphate buffered saline (PBS), fixed by methanol and stained by hematoxylin (HHSM, Accustain, Sigma-Aldrich) for 5 min. The remaining cells were removed from the upper side of the membrane by scrubbing. Quantification of the migration assay was performed by counting the number of cells at the lower surface of the filters (20 different fields per condition).

### Cell Viability

Cell viability was assessed by using 3-(4,5-dimethyl-2-thiazolyl)-2,5-diphenyl-2H-tetrazolium bromide (MTT, Sigma-Aldrich, Inc.). MTT was solubilized in culture media without FCS at the final concentration of 0.5 mg/mL. Cells were incubated with MTT for 50 min at +37°C in the dark. The purple formazan crystals produced by viable cells were dissolved by DMSO and the absorbance was quantified at 550 nm with an Infinite^®^ 200 Pro reader (Tecan Trading AG).

### Electrophysiology

Magnesium Inhibited Cation (MIC) currents were recorded using the whole-cell patch-clamp technique as previously published (Rybarczyk et al., 2017). The composition of the extracellular solution was (in mM): Na-gluconate 150; K-gluconate 5; Mg-gluconate 2; Ca-gluconate 2; HEPES 10; glucose 5; TEA-Cl 5; pH adjusted to 7.4 with NaOH. The composition of intrapipette solution was (in mM): Na-gluconate 8; Cs-methanesulfonate 145; EGTA 10; HEPES 10; pH adjusted to 7.2 with CsOH. Membrane potential was held at −40 mV and currents were elicited by a ramp depolarization from −100 mV to +100 mV for 350 ms at the frequency of 0.1 Hz. Signals were filtered at 1 kHz and digitized at 5 kHz using an Axopatch 200B patch-clamp amplifier (Molecular Devices, Sunnyvale, CA, United States) combined with a 1322A digidata (Molecular Devices, Sunnyvale, CA, United States). MIC currents developed during the dialysis of the intracellular media by a free Mg intrapipette solution (Prakriya and Lewis, 2002). Membrane currents were expressed as current densities in pA/pF. All experiments were performed at room temperature.

### Cell Transfection

Cell transfections with siRNA were performed as previously described (Rybarczyk et al., 2012). The TRPM7 siRNA used in the current study (5′-GTCTTGCCATGAAATACTC-3′) targets the mRNA sequence coding for the 170–188th N-terminal region of TRPM7 and was previously proved to be an effective target for TRPM7 silencing (Hanano et al., 2004; Vanlaeys et al., 2020). SiRNA were transfected in pancreatic cancer cells by nucleofection using a NucleofectorTM II device (Lonza, Bâle, Switzerland). Cells (10^6^ cells) were transfected with 2 μg siRNA (corresponding to a final concentration of 1.5 μM) according to the optimized protocol recommended by Lonza. All the experiments were performed 48 h after the nucleofection. In parallel, cells were transfected with a non-targeting siRNA (siControl). Non-targeting and TRPM7-targeting siRNA were both provided by Dharmacon Research Inc., United States.

### Quantitative RT-PCR

Total RNA isolation, reverse transcription and real-time PCR analysis were performed as previously described (Rybarczyk et al., 2017).

### Western Blotting

Cells were lysed 30 min on ice in RIPA assay buffer (1% Triton X-100, 1% Na deoxycholate, 150 mM NaCl, 10 mM PO4Na2/K, pH 7.2) supplemented with Sigma P8340 inhibitors cocktail, 2 mM EDTA, and 5 mM orthovanadate. After centrifugation at 13000 rpm, the proteins in the supernatant were quantified using the BCA method (BioRad). Equal amounts of each protein sample (50 μg) were separated by electrophoresis on sodium dodecyl sulfate (SDS) polyacrylamide gel electrophoresis and blotted onto nitrocellulose membrane (Amersham). Blots were incubated with antibodies raised against TRPM7 (1/1000, ab109438, Abcam) and GAPDH (1/4000, ab8245, Abcam). Blots were developed with the enhanced chemiluminescence system using specific peroxidase-conjugated anti-IgG secondary antibodies.

### Confocal Microscopy

Cells were plated on glass slides and incubated in 10% serum-containing medium for 16 h. AG-9 synthetic elastin peptides (10^–7^ M) were then added to serum-free culture medium supplemented or not with EGCG 10 μM and cells were incubated at +37°C for 24°C. After several washes, cells were fixed for 5 min with 4% paraformaldehyde. The slides were washed with PBS-T and saturated in PBS-T with 5% BSA. Cells were then incubated for 1 h at room temperature with the first antibodies diluted 1/400 in PBS-T with 1% BSA. Slides were washed in PBS-T and cells were incubated for 30 min with the Alexa-488 or Alexa-568-conjugated secondary antibodies diluted 1/1000 in PBS-T with 1% BSA. Cells were then washed with PBS-T. Control preparations were incubated with omission of the first antibody. Immunofluorescence-labeled cell preparations were studied using a Zeiss LSM 710^®^ NLO confocal laser scanning microscope (Carl ZEISS SAS, Marly-le-Roi, France) with the 63× oil-immersion objective (ON 1.4) coupled with CHAMELEON femtosecond Titanium-Sapphire Laser (Coherent, Santa Clara, CA, United States). Alexa 488 and 568 were sequentially excited by 488 nm line of Argon laser and diode laser 561 nm. Emitted signals were, respectively, collected with 493–560 nm and 570–700 bandpass filters. Image acquisitions were performed with ZEN Software (Carl ZEISS SAS, Marly-le-Roi, France) and all acquisition settings were constant between specimens. Colocalization analyses were made with ImageJ software (Colocalization Analysis plugin).

### Immunohistochemistry

Human tissues samples from PDAC (*n* = 8) were used with the agreement of patients treated by surgery in the University Hospital of Amiens (Picardie, France). Experiments on human tissues were approved by the Comité Consultatif de Protection des Personnes dans la Recherche Biomédicale de Picardie (Amiens, France). Immunohistochemistry was performed on human tissues using the indirect immune-peroxidase staining technique on the paraffin-embedded material with a Ventana XT instrument (Ventana Medical Systems, Roche Diagnostics). Tissue sections were obtained from 8 patients undergoing a surgery of PDAC at Amiens hospital, France. The 8 cases of PDAC were diagnosed as moderately differentiated by confirmed pathologists.

Each selected section contained both tumoral and non-tumoral adjacent pancreatic tissues. Sections were incubated with anti-RPSA Rabbit polyclonal antibody (ab99484 from Abcam), and negative controls were performed after deparaffinization with xylene and dehydration with a serial ethanol gradient. Antigens were retrieved by heating slides in citrate buffer (pH 6.0) for 8 min at +95°C. Samples were incubated for further 56 min with Ultra CC2 (cell conditioning, pH 6.0) and anti-RPSA antibody (diluted 1/2000) was applied for 32 min. Negative control was realized by omitting the primary antibodies. Analysis of tissue section was done after counter-coloration by light microscopy by confirmed pathologists (PR and DC).

### Statistical Analysis

Data are presented as mean ± SEM and n refers to the number of independent repeat of experiment. Statistical analyses were made using Student’s *t*-tests or Mann-Whitney rank sum test depending on sample normality or paired Wilcoxon signed rank test using Sigma-Stat 3.0 (Systat Software, Inc.). When more than two conditions were compared, a Kruskal–Wallisone-way ANOVA was used followed by *post hoc* Dunn’s Method tests. Results were considered significant when *P < 0.05, **P < 0.01, and ***P < 0.001.

## Results

### Chronic Treatment With AG-9 and VG-6 Increases Pancreatic Cancer Cell Migration Through TRPM7 Expression

**FIGURE 1:**
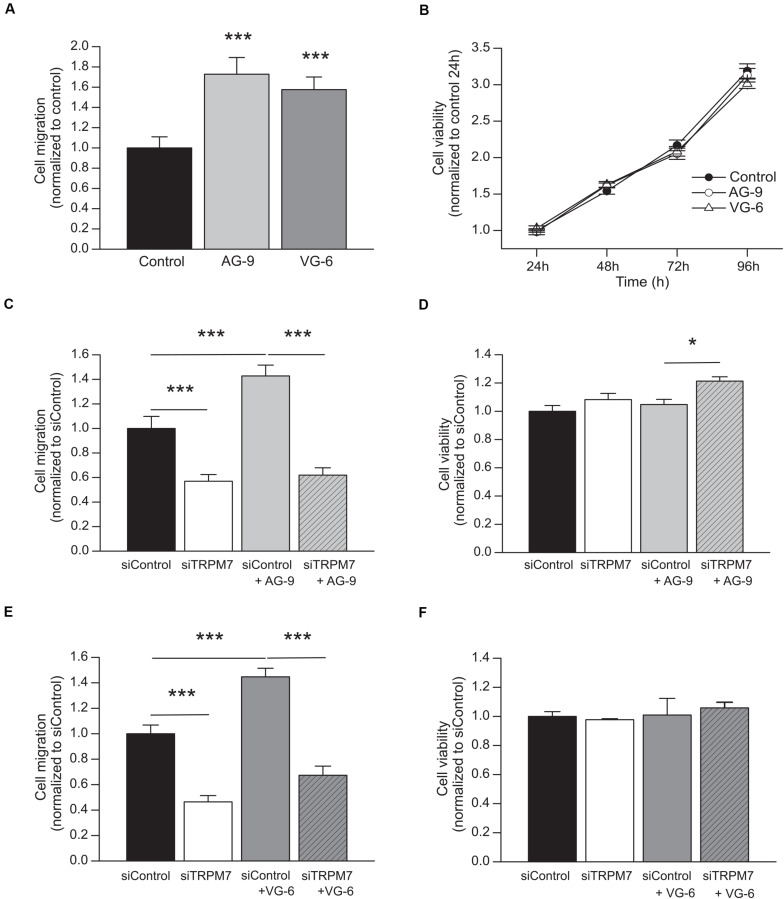
EDPs enhance MIA PaCa-2 migration through TRPM7 expression. **(A)** Effect of 24 h incubation with 10^–7^ M AG-9 and VG-6 EDPs on the MIA PaCa-2 cell migration assessed by Boyden chamber assay. **(B)** Effect of EDPs on cell viability assessed by MTT assay. **(C)** Effect of TRPM7 silencing on AG-9 enhanced cell migration. **(D)** Effect of TRPM7 silencing and AG-9 treatment on cell viability. **(E)** Effect of TRPM7 silencing on VG-6 enhanced cell migration. **(F)** Effect of TRPM7 silencing and VG-6 treatment on cell viability. All results are shown as means ± SEM. *P < 0.05; ***P < 0.001 by ANOVA followed by *post hoc* Dunn’s tests.

MIA PaCa-2 cells were incubated with AG-9 or VG-6 at 10^–7^ M for 24 h. Cell migration was increased by 72.8 ± 16.5% for AG-9 (*n* = 4) and by 57.6 ± 12.5% for VG-6 (*n* = 4) (*P* < 0.001; Figure 1A). Cell viability was assessed during 96 h and no effect of EDPs was observed (Figure 1B). These results showed that EDPs increased PDAC cell migration without changing the cell viability. We previously showed that PDAC cell migration is dependent of TRPM7 expression (Rybarczyk et al., 2012, 2017). We confirmed that TRPM7 was implicated in MIA PaCa-2 basal migration since cell migration was reduced in cells transfected with a siTRPM7 (*n* = 4; *P* < 0.001; Figures 1C,E). AG-9 and VG-6 had still a pro-migratory effect in cells transfected with a scrambled siRNA but they had no effect in siTRPM7 cells (*n* = 4; *P* > 0.05; Figures 1C,E). MTT assays were also performed to control that the effects observed for cell migration were not due to modifications of cell viability. AG-9 treatment slightly increased cell viability by 21.33 ± 3.05% only in cells transfected with a siRNA targeting TRPM7 (*n* = 4; *P* < 0.05; Figure 1D). On the other hand, VG-6 treatment had no effect on cell viability (*n* = 3; *P* < 0.05; Figure 1F). Taken together, our results show that EDPs increased PDAC cell migration in a TRPM7 dependent manner.

### AG-9 Stimulates TRPM7 Currents in Pancreatic Cancer Cells

**FIGURE 2:**
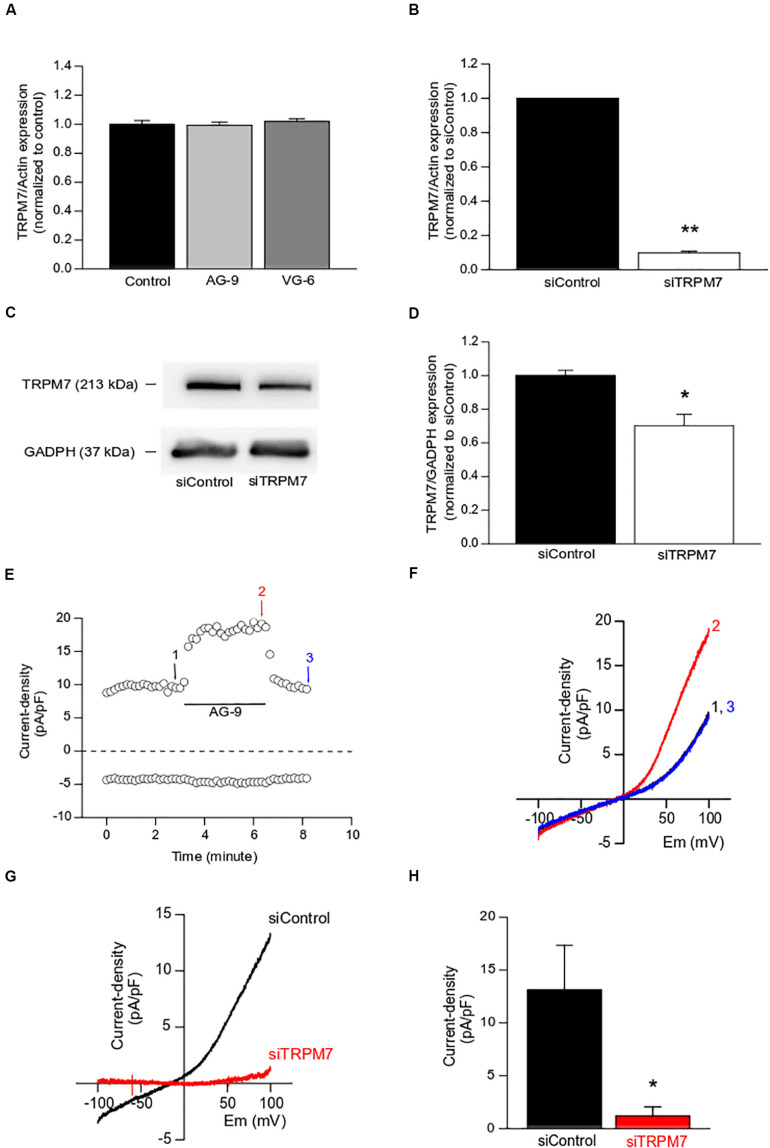
Functional modulation of TRPM7 channels by EDPs. **(A)** Effect of EDPs incubation on TRPM7 expression assessed by qRT-PCR in MIA PaCa-2 cells. **(B)** Effect of TRPM7 silencing on TRPM7 expression assessed by qRT-PCR. **(C)** Typical example of lysates from MIA PaCa-2 cells transfected with a scrambled siRNA (siControl) or targeting TRPM7 (siTRPM7) and immunoblotted with anti-TRPM7 and anti-GADPH antibodies. **(D)** Quantification of immunoblotting normalized to siControl. **(E)** Typical example of MIC current recorded at +100 mV (outward current) and at −100 mV (inward current) before (1), during (2), and after (3) the application of AG-9 (10^–7^ M). **(F)** Current–voltage relationships corresponding to the traces recorded in **(C)**. **(G)** Averaged current–voltage relationship of AG-9-activated currents recorded in cells transfected with a non-targeting siRNA (siControl, black traces) and in cells transfected with a siRNA targeting TRPM7 (siTRPM7, red traces). **(H)** Current densities of AG-9-activated currents recorded at +100 mV. *P < 0.05 and **P < 0.01 by Mann-Whitney rank sum tests.

Firstly, TRPM7 expression was assessed by quantitative RT-PCR following the treatment with AG-9 or VG-6 for 24 h. TRPM7 expression was not modified by EDP treatment (*n* = 4; *P* < 0.05; Figure 2A) while TRPM7 silencing decreased TRPM7 expression by 90.2 ± 0.1% at mRNA level (*n* = 4; *P* < 0.01; Figure 2B), and by 30 ± 7% at protein level (*n* = 4; *P* < 0.05; Figures 2C,D) when compared to siControl. We previously showed that Magnesium-Inhibited Cation (MIC) currents are mainly due to TRPM7 channel activity in MIA PaCa-2 cells (Rybarczyk et al., 2017). MIC currents were recorded by using the conventional technique of patch-clamp in whole-cell configuration. Maximal MIC current intensity was reached after almost 15 min of intracellular media dialysis with EGTA (data not shown, see Rybarczyk et al., 2017). A typical example of AG-9 acute perfusion effect on MIC currents is displayed in the Figures 2E,F. AG-9 increased outward (recorded at +100 mV) in a sustained and reversible manner (representative trace of 5 experiments, Figure 2E). I-V relationships showed that AG-9 increased the outward rectification (representative trace of 5 experiments, Figure 2F). We further built the I-V relationships of AG-9 sensitive currents by subtracting the basal MIC current from that recorded during AG-9 perfusion. AG-9-sensitive currents had inward component at negative membrane potentials, strong outward rectification at positive potential and a reversal membrane potential close to 0 mV (*n* = 5; Figure 2G). Interestingly, the AG-9-sensitive currents seem more linear than the typical MCI currents. TRPM7 silencing fully abolished the AG-9-sensitive currents indicating that AG-9 activated TRPM7 channels in PDAC cells (*n* = 4; *P* < 0.05; Figures 2G,H).

### TRPM7 and RPSA Colocalize in Pancreatic Cancer Cells

**FIGURE 3:**
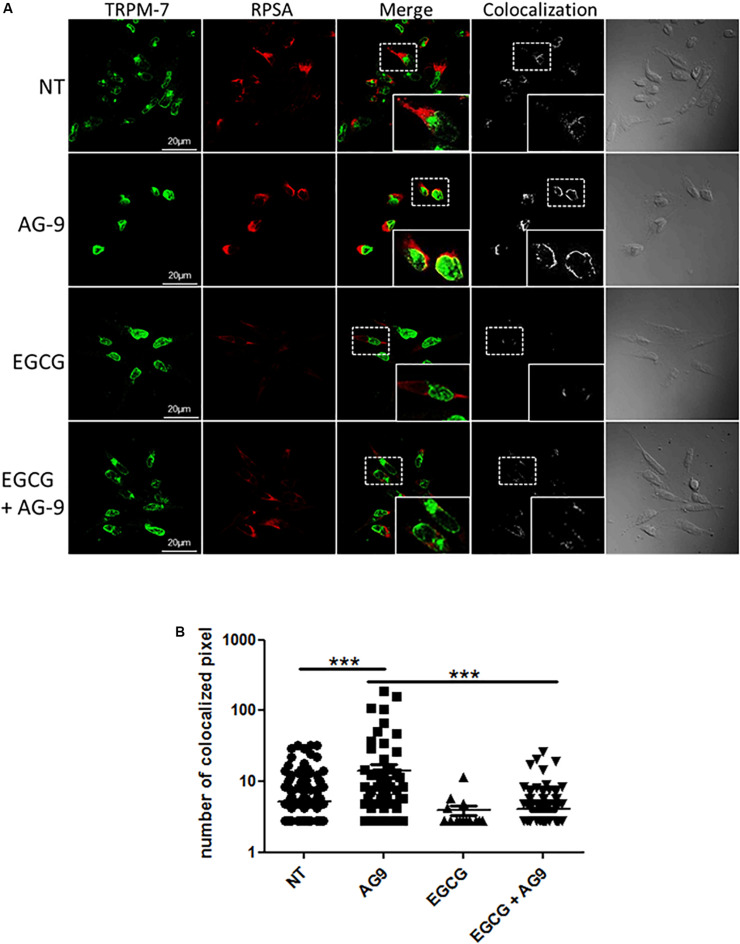
Cellular distribution of TRPM7 and RPSA. **(A)** MIA PaCa-2 cells were pre-incubated with or without EGCG (10 μM) for 1 h and then with or without AG-9 (10^–7^ M) for 24 h at +37°C and analyzed by confocal microscopy for TRPM7 and RPSA protein cellular distribution. Colocalization was studied with the Colocalization plugin of ImageJ. Inserts: 2.25× magnification. Scale bar: 20 μm. **(B)** Quantification of TRPM7/RPSA colocalization pixels in confocal optical sections of MIA PaCa-2 cells in the presence or not of AG-9 (10^–7^ M) and EGCG (10 μM). Data from one experiment, representative of three independent experiments, are shown. ***P < 0.001 by Mann-Whitney rank sum tests.

We previously showed that EGCG treatment prevents AG-9-induced blebbing by RPSA inhibition (Brassart et al., 2019). As demonstrated above, AG-9 activates TRPM7 channels. We have previously shown that AG-9 interacts with cancer cells through RPSA binding. We were interesting in the possible relation between RPSA and TRPM7. EGCG was previously reported to bind RPSA and to prevent AG-9/RPSA interaction. For this reason, MIA PaCa-2 pancreatic cancer cells were pre-incubated with EGCG (10 μM) for 1 h, then incubated with or without AG-9 (10^–7^ M) for 24 h before fixation with paraformaldehyde and labeling with anti-TRPM7 and RPSA antibodies. Immunocytofluorescence microscopy analysis on optical sections showed TRPM7/RPSA colocalization in absence of effectors. In presence of AG-9 peptide, TRPM7/RPSA colocalization increases by 269 ± 61% (*n* = 3; *P* < 0.001; Figures 3A,B). TRPM7/RPSA colocalization was not significantly modified by EGCG treatment in comparison with untreated MIA PaCa-2 cells. Co-treatment with AG-9 and EGCG significantly decreased TRPM7/RPSA colocalization by 71.7 ± 1.4% (*n* = 3; *P* < 0.001; Figures 3A,B). These data demonstrated that AG-9 peptide influences the colocalization of TRPM7 and RPSA in pancreatic cancer cells.

### RPSA Is Overexpressed in Human PDAC Tissues

**FIGURE 4:**
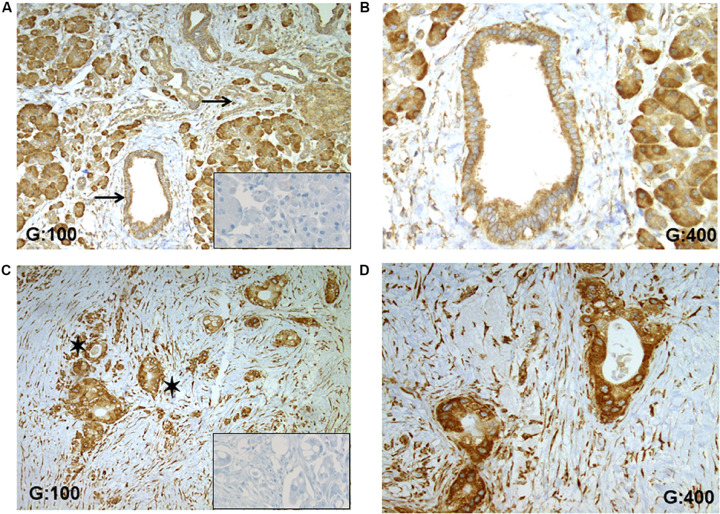
RPSA expression in human PDAC. **(A)** RPSA is ubiquitously expressed in the normal pancreatic tissue (pancreatic duct and acinar cells, inflammatory, and stromal cells) but no unspecific staining was seen in collagen fibers, black arrows focus on healthy pancreatic ducts. **(B)** At high magnification, a weak and cytosolic staining was observed in normal duct cells. **(C)** In PDAC tissue, a high staining was observed in all tumoral cells, and black stars show tumoral glandular structures. **(D)** At high magnification, a high and cytosolic staining was always observed in tumoral cells. Inserts: RPSA staining was not apparent in the absence of the primary antibody.

The expression of RPSA in human PDAC tissues was studied using IHC and demonstrated a higher expression in tumoral cells compared to non-tumoral duct cells (Figure 4). In the 8 selected patients suffering of a moderately differentiated PDAC, a strongest staining was recorded in tumoral tissues compared to non-tumoral pancreatic ducts (Figures 4A,C). For each case, the staining was localized into the cytoplasm with little variation dependent of the characteristic of tumoral cells (hypersecretion, microvacuoles in the cytoplasm). In non-tumoral tissues into ducts, the staining was also cytoplasmic and RPSA seems to be ubiquitously expressed (Figure 4A). Indeed, other cell types like inflammatory, stromal and acinar cells showed a specific staining. Importantly, no unspecific staining was observed, especially in extracellular matrix fibers like collagen (Figure 4B).

Focusing on pancreatic ducts, an overexpression of RPSA was observed in tumoral cells. Anti-RPSA staining was stronger in tumoral cells (Figure 4D) compared to the non-tumoral duct cells (Figure 4C).

## Discussion

In the present study, we showed that TRPM7 is involved in the MIA PaCa-2 cell migration stimulated by elastin-derived peptides (EDPs) AG-9 and VG-6 and that EDPs treatment lead to TRPM7 / RPSA interaction in PDAC cells.

These results confirm that EDPs exert protumor activities by increasing cell migration as previously shown by Da Silva et al. (2018) in HT-29 colon adenocarcinoma cell line. Pancreatic adenocarcinoma (PDAC) is characterized by an abundant desmoplastic stroma composed by extracellular matrix (ECM) proteins and various cell types. The ECM represents up to 90% of the PDAC tumor mass. For instance, it has been shown that collagen I and IV promote PDAC cell proliferation and migration (Lafaro and Melstrom, 2019). Thus, our results provide new insights into the regulation of PDAC cell migration by ECM. To our knowledge, the role of EDPs had not yet been described in PDAC. Our work suggests that EDPs could participate to the stimulation of PDAC cell migration and invasion induced by the interaction with the desmoplastic stroma.

EDP-stimulated cell migration was prevented by TRPM7 silencing indicating that this protein is required for this mechanism. TRPM7 is overexpressed in numerous malignancies including PDAC (Rybarczyk et al., 2012; Yee et al., 2015). *In vitro*, TRPM7 silencing reduced basal (non-stimulated) migration (Rybarczyk et al., 2012) and basal or FBS-stimulated PDAC cell invasion (Yee et al., 2015; Rybarczyk et al., 2017). EDP treatment did not modify TRPM7 expression but acute application of EDP induced the generation of a large TRPM7-like membrane current in MIA PaCa-2 cells. TRPM7 channels are essential for Ca^2+^, Mg^2+^, and Zn^2+^ cellular influx (Mittermeier et al., 2019). It has been shown that EDPs increases cytosolic calcium levels in human fibrosarcoma cells (Brassart et al., 2019). Surprisingly, we did not observe any effect of chronic nor acute application of EDPs on cation influx recorded by manganese-induced quenching of fura-2 fluorescence (data not shown). These results suggest that EDPs activated TRPM7 channels without inducing a large increase of divalent cation influx in the cytosol. However, we cannot exclude that EDPs induced variation of divalent cation concentration into highly localized nanodomains. For example, TRPM7 channels are linked to high-calcium microdomains, also called calcium flickers or sparks, promoting directional migration in human lung fibroblasts (Wei et al., 2009) and also invadosome formation in mouse neuroblastoma cells (Visser et al., 2013). TRPM7 is a non-selective cation channel fused with a functional kinase domain at its c-terminus (Nadler et al., 2001; Runnels et al., 2001). Several studies also showed a role of TRPM7 kinase domain in cancer cell migration (Middelbeek et al., 2012; Guilbert et al., 2013; Song et al., 2017). Interestingly, the AG-9 sensitive currents seem more linear than the typical MIC currents. Kozak et al. (2002) described that external Mg^2+^ blocked monovalent cation current in a fast, reversible and voltage-dependent manner. Our data suggest that AG-9 only increased the monovalent component of TRPM7 current and particularly the outwardly rectifying one. It is tempting to speculate that AG-9 interferes with external Mg^2+^ to change TRPM7 permeation. Taken together, these data strongly suggest that AG-9 modifies monovalent but not divalent currents through TRPM7. However, this is only descriptive and further experiments are needed to better understand how AG-9 acts with TRPM7 channels to enhance pancreatic cancer cell migration.

We recently showed that EDPs induced cancer cell blebbing and shedding of extracellular vesicles through binding to RPSA (Brassart et al., 2019). Here, we further showed that EDPs treatment induced the colocalization of TRPM7 and RPSA in MIA PaCa-2 cells. This colocalization was prevented by co-treatment with AG-9 and EGCG, an inhibitor of RPSA. Taken together, our results show that EDPs stimulate PDAC cell migration and TRPM7/RPSA colocalization. Interestingly, it has been shown that RPSA interacts with integrin alpha 6 (ITGA6) and regulates PDAC cell invasion through MAPK signaling pathways (Wu et al., 2019). TRPM7 silencing reduced the phosphorylation level of MAPK signal molecules (P38, ERK, and JNK) in metastatic breast cancer cells and decreased their migration and invasion (Meng et al., 2013). Based on our results and the literature, we can hypothesize that EDP release in the desmoplastic stroma during pancreatic carcinogenesis may induce formation of TRPM7/RPSA complexes in PDAC cells. It is tempting to speculate that such complexes may activate oncogenic signaling pathways leading to enhanced cell migration but this hypothesis needs further investigations. Moreover, our results confirm that RPSA is overexpressed in human pancreatic tumor tissues compared to their adjacent non-tumor counterparts (Wu et al., 2019). RPSA is ubiquitously expressed and IHC staining was observed in number cell types without any unspecific staining. This ubiquitous expression need to focus on non-tumoral ducts cells and to compare them to the tumoral cells. Wu et al. (2019) described the same overexpression of RPSA in PDAC tissues by using different antibodies. Interestingly, we previously showed a similar overexpression of TRPM7 in PDAC primary tumor (Rybarczyk et al., 2012) as well as in lymph node (Rybarczyk et al., 2017). Further investigations are needed to increase the number of patients and the diversification of tumor status (grading, metastatic status, …). Thus, targeting of TRPM7/RPSA complexes could be a promising strategy to reduce cancer cell migration in the neoplastic pancreas.

## Data Availability Statement

The raw data supporting the conclusions of this article will be made available by the authors, without undue reservation, to any qualified researcher.

## Ethics Statement

The studies involving human participants were reviewed and approved by human tissue samples from PDAC (*n* = 8) and were used with the agreement of patients treated by surgery in the University Hospital of Amiens (Picardie, France). Experiments on human tissues were approved by the Comité Consultatif de Protection des Personnes dans la Recherche Biomédicale de Picardie (Amiens, France). The patients/participants provided their written informed consent to participate in this study.

## Author Contributions

TL did the experiments (cell culture, treatments, cell migration, and proliferation assays). PR and DC did the experiments (IHC in patient tissues). AV did the experiments (Western-Blots). CB and RC helped for the cell treatment. SB-P and ID-D helped to design the experiments (confocal microscopy for SB-P and IHC for ID-D). HO-A helped to design the experiments and correct the manuscript. BB and MG designed the study, wrote the manuscript, and did the experiments (confocal microscopy for BB and patch-clamp for MG). All authors contributed to the article and approved the submitted version.

## Conflict of Interest

The authors declare that the research was conducted in the absence of any commercial or financial relationships that could be construed as a potential conflict of interest. The handling editor declared a past co-authorship with one of the authors SB-P.
